# Investigating the Effects of UVC Exposure at the Limbus

**DOI:** 10.3390/cells15110967

**Published:** 2026-05-23

**Authors:** Bethany P. Torr, Jennifer P. Craig, Simon J. Dean, Trevor Sherwin, Sanjay Marasini

**Affiliations:** 1 Department of Ophthalmology, Aotearoa New Zealand National Eye Centre, The University of Auckland, Private Bag 92019, Auckland 1142, New Zealandt.sherwin@auckland.ac.nz (T.S.); s.marasini@auckland.ac.nz (S.M.); 2 Department of Optometry and Vision Science, Aotearoa New Zealand National Eye Centre, The University of Auckland, Auckland 1142, New Zealand

**Keywords:** antimicrobial effects, cyclobutane pyrimidine dimers, corneal transmissibility, limbal stem cell safety, ultraviolet C light penetration

## Abstract

**Highlights:**

**What are the main findings?**

- Low-intensity UVC effects on DNA at the limbus remain limited to superficial epithelial corneal layers, irrespective of dose.

**What are the implications of the main findings?**

- Limited UVC penetration based on depth of detectable CPD formation suggests sparing of limbal stem cells from DNA damage.
- Demonstrates potential safety of therapeutic low-intensity corneal UVC application in terms of CPD formation.

**Abstract:**

**Purpose:** Preclinical studies report low-intensity ultraviolet C (UVC) light to be safe and effective in treating murine bacterial keratitis, however, limbal impacts of UVC have yet to be investigated directly. This study evaluated the depth and density of UVC-induced DNA damage in the porcine and human limbus following UVC exposures of varying supratherapeutic dose. **Methods:** The corneoscleral junction (limbus) of full-thickness porcine corneas was exposed to supratherapeutic doses of UVC light (265 nm, 1.93 mW/cm^2^) for 5, 10, 15, 30, or 60 min (exposure groups) or remained unexposed for the same durations (control groups), with a sample size of 6 per group. In parallel, human corneal tissue was exposed to UVC for 1 or 5 min and processed identically. Following exposure, all tissues were frozen, dissected, and analysed using immunohistochemistry to detect cyclobutane pyrimidine dimers (CPDs) as markers of DNA damage. CPD distribution, depth, and density were subsequently evaluated. **Results:** CPDs were localised predominantly in the superficial corneal epithelial layers, irrespective of the UVC dose. The mean ± SD thickness of the corneal epithelium in the UVC-exposed groups was 38.9 ± 18.9 µm, and the average depth of CPD formation was 13.3 ± 8.43 µm. The proportions of cells affected by CPDs within the corneal epithelium (mean ± SD) were 47.8 ± 25.6%, 58.5 ± 16.2%, 39.9 ± 26.4%, 41.3 ± 27.3%, and 38.9 ± 28.3% for exposure durations of 5, 10, 15, 30, and 60 min, respectively (*p* > 0.05). Human cornea showed similarly limited penetration, with no difference in CPD proportions between the 1 and 5 min UVC exposures (*p* = 0.70). **Conclusions:** UVC-induced DNA damage in both species was confined to the superficial cellular layers of the cornea, with no detectable damage observed in deeper tissues, including those where limbal stem cells reside, even after supratherapeutic doses of up to one hour of exposure.

## 1. Introduction

Low-intensity ultraviolet C (UVC) light has been studied as a promising therapeutic approach for treating superficial corneal infections [1,2]. Preclinical studies have demonstrated the safety of UVC in managing corneal infections, with treatment durations as short as 15 s twice daily for three days proving effective (90 s cumulative dose) [1,3,4,5]. UVC exposure induces photochemical reactions in DNA, leading to the formation of mutagenic photoproducts, such as cyclobutane pyrimidine dimers (CPDs) and pyrimidine(6–4)pyrimidone photoproducts [(6–4)PPs], which disrupt DNA replication and trigger cell death [4]. UVC is highly effective at targeting bacterial cells while causing minimal damage to corneal tissue [4]. While it is well established that UVC is rapidly absorbed by superficial cell layers at the central cornea, its capacity to penetrate deeper layers more peripherally and affect cells like limbal stem cells remains uninvestigated [2]. This is important as significant insults to the limbus such as alkali burn can result in limbal stem cell failure and subsequent corneal opacity [6].

A recent study demonstrated that the penetration depth of UV light in the rat corneal limbal epithelium varies according to wavelength, similar to its behaviour in the central corneal epithelium [7]. Specifically, UVB (311 nm) and UVC (254 nm) wavelengths were able to reach the basal cells, whereas UVC at 235 nm penetrated only as far as the middle epithelial cell layers. In porcine corneas exposed to 254 nm UVC, CPD-positive cells were detected in the superficial layers and extended up to 50–100 µm deep, but not into the basal region. Given that the porcine cornea is thicker and shares similarities with the human cornea, these findings would suggest that irradiation with 265 nm light at the limbus may be safe. However, this hypothesis has yet to be experimentally confirmed [8,9]. UVC penetration at the limbus could pose a risk to stem cells and potentially interfere with corneal wound healing [10]. Therefore, confirming the safety of UVC exposure in the limbal region is of critical importance.

Given existing evidence that UVC light is rapidly absorbed by biological tissues, it is hypothesised that increasing the UVC dose will not significantly enhance its penetration depth through the cornea [1,11]. Therefore, the aim of this study was to evaluate UVC penetration through limbal tissue, with a particular focus on DNA damage at exposure levels exceeding those required for keratitis management. To ensure greater clinical relevance, this study was conducted on ex vivo full thickness corneal tissue rather than cultured cells, as whole tissue samples provide a more accurate representation of in situ conditions [12]. The depth and density of UVC-induced DNA damage in porcine corneas was assessed, and the findings were validated in human donor corneas. While well-chosen animal models can effectively simulate biological effects on human tissues, it is essential to test these outcomes in environments that closely mirror human clinical settings to facilitate a smooth translation from preclinical research to clinical application [13].

## 2. Methods

### 2.1. Light Source

The light source in these experiments is a 265 nm wavelength UVC diode with a fixed intensity of 1.93 mW/cm^2^ that projects a spot size of 4.5 mm diameter at a distance of 8 mm, as described previously [1]. Treatment durations were controlled using an electronic timer (dose = intensity × exposure time).

### 2.2. Assessment of the Depth of UVC Penetration Through Porcine Cornea

Depth of UVC penetration through the cornea was evaluated by quantifying CPD formation observed using immunohistochemistry. Fresh porcine eyes were obtained from a local slaughterhouse, thoroughly cleansed, and examined for epithelial integrity. Eyes without an intact cornea during a visual inspection were excluded. A 6 mm diameter section of the cornea at the corneoscleral junction (limbus) was excised using a biopsy punch. Multiple corneal buttons were harvested from each eye and randomly assigned to 10 case and control groups, ensuring that each eye was represented across all experimental conditions. A sample size of 6 was used consistently across all 10 groups. This was based on conventional practice and informed by the group’s previous studies [2], which demonstrated a clear and low-variability difference between cases and controls. The corneal buttons were then exposed to UVC for durations of 5, 10, 15, 30, or 60 min (sample size of 6 for each exposure), while control samples were left unexposed for the same lengths of time (sample size of 6 each). Tissues were subsequently frozen in Tissue-Tek^®^ OCT (ProSciTech, Kirwan, Australia) and sectioned into 18 μm slices parallel to the optical axis of the eye using a Micron HM550 cryostat (Thermo Fisher Scientific, Waltham, MA, USA). The prepared slides were stored at −20 °C for further analysis.

### 2.3. Immunohistochemistry Analysis

Slides were rinsed twice with PBS for 10 min each to remove Tissue-Tek^®^ OCT. The tissues were then permeabilised for 30 min with 0.5% Triton X-100 (Merck Life Science Pty Ltd., Melbourne, VIC, Australia) and washed twice with PBS for 10 min. To prevent non-specific binding, tissues were incubated at room temperature for 1 h in 250 µL of 10% Normal Goat Serum (NGS) with 0.2% Triton X-100 in PBS. They were then incubated overnight at 4 °C with mouse anti-thymine dimer primary antibody (ab10347, 1:2500; Abcam Inc, Melbourne, Australia) in an immuno buffer of 10% NGS and 1% Triton X-100 in PBS. The following day, slides were washed three times with PBS over 10 min and incubated in the dark at room temperature for 2 h with the secondary antibody (goat anti-mouse Cy3, 115-165-003, 1:500, Invitrogen, Carlsbad, CA, USA) in 0.2% Triton X-100 in PBS. After rinsing three times with PBS for 10 min, slides were incubated for 10 min with DAPI (1:1000) in the dark for nuclei staining. Slides were rinsed twice with PBS for 5 min, air dried, treated with Citiflour (ProSciTech, Kirwan, Australia), covered with a coverslip, sealed with nail polish, and stored in the dark at 4 °C until imaging. Imaging was conducted on an Olympus FV1000 confocal laser scanning microscope (Olympus, Tokyo, Japan) using FV-10 ASW 3.0 Viewer, with Cy3 and DAPI (Merck Life Science Pty Ltd., Melbourne, Australia) excited by 532 nm and 405 nm lasers, respectively, and detected using band-pass emission filters at 570 nm and 461 nm. Voltage and offset settings were optimised to prevent oversaturation and enhance labelling discrimination and remained constant across all repeats. Non-specific secondary antibody binding was noted in the secondary-only control and across all exposure groups.

### 2.4. Assessment of the Depth of UVC Penetration Through Human Donor Cornea

Corneal rims from three male human donors (aged 48, 83 and 26 years) were obtained from the NZ National Eye Bank, with approval from the Health and Disability Ethics Committees (Ethics reference: NTX/07/08/080/AM04). Sample size was therefore based on convenience sampling, based on the availability of the donor tissue. Six biopsy punches, 6 mm diameter each, were extracted from each corneal rim at the limbal region, and the resulting rims were randomised into groups for 1 min control, 1 min UVC exposure, 5 min control, and 5 min UVC exposure (minimum sample size of 5 for each group). Biopsy punches were either exposed to UVC for 1 or 5 min or left unexposed for equivalent durations. The selected exposure times were based on the consideration that therapeutic UVC application for keratitis management will not exceed a cumulative duration of 5 min. Following experimental exposures, the tissues were frozen in Tissue-Tek^®^ OCT, sectioned into 18 μm slices parallel to the optical axis of the eye using a Microm HM550 cryostat (Thermo Fisher Scientific, Kalamazoo, MI, USA), and analysed using immunohistochemistry as before.

### 2.5. ImageJ Quantification Within Corneal Epithelium

Images (unmasked) were imported to ImageJ (National Institute of Health, Bethesda, MD, USA; version 1.54), the limits of the corneal epithelium were marked (region of interest) from the most superficial visible boundary to the deepest visible boundary, using the freehand selection tool, and the rest of the structures were removed. Images were then split into red, green, and blue channels. The red channel represented CPD defects and the blue channel represented cell nuclei (DAPI-stained), whereas no data were captured in the green channels and thus the values were zero. Pixels which contain significant levels of blue and red data appear as pink in the images. The images were subsequently enhanced in PowerPoint using consistent settings across all images to improve brightness and contrast, to optimise visualisation of the cells.

**Figure 1:**
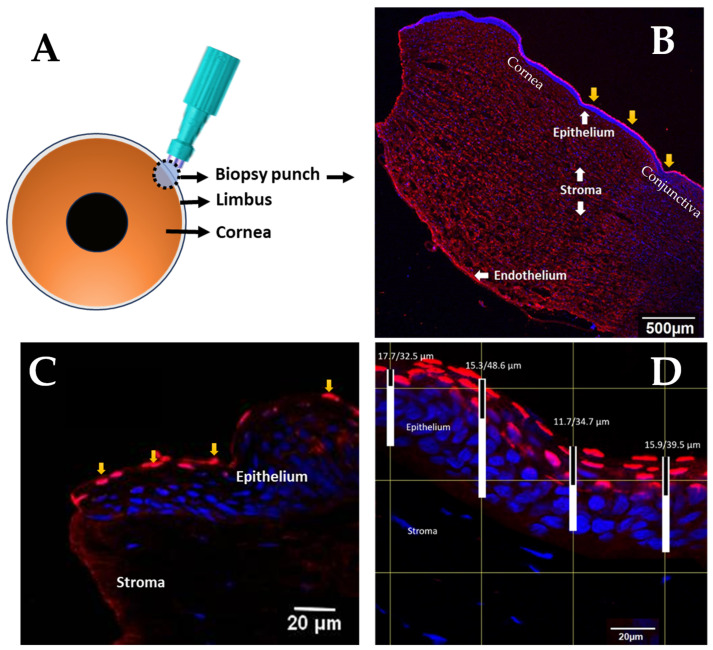
Methods used to detect UVC-induced DNA defects in the porcine cornea. Schematic diagram of porcine cornea and biopsy punch of the limbal region is shown in (**A**). The biopsied limbal tissues were dissected and labelled for CPD using immunohistochemistry (**B**). Low magnification (4× objective) was used to capture the entire sample—yellow arrows represent cells with DNA defects overlying normal corneal epithelium (blue stain). The limbal region with superficial CPD-positive cells (yellow arrows) is shown in (**C**) (60× objective). Corneal epithelial thickness following UVC exposure was measured using ImageJ software (**D**). Rectangular grids were laid over confocal microscopy images (20× objective), and the depth of the limbal epithelium (white bar) and CPD-positive cells (black bars) was assessed at four grid points that defined the upper and lower boundaries of the epithelium. Blue and pink cells represent normal cells and cells with DNA photoproducts, respectively. Non-specific staining is visible in the stroma (**B**,**C**).

Cell counting for both control and UVC-exposed groups utilised the blue channel, which highlighted the total number of DAPI-stained nuclei. Since corneal epithelial cells are mononuclear, nucleus count was used to represent cell count. For CPD counts, red and blue channels were overlaid, resulting in pink cells that enhanced quantification accuracy by differentiating damaged nuclei (pink) from non-specific antibody binding (red). A single observer manually counted the cells using the multi-point tool in ImageJ. To minimise potential bias, image acquisition and image analyses were carried out independently by two individuals (SM and BT). The trained individual performing image acquisition was masked from the image analysis, and vice versa. Epithelial thickness measurements were taken from the overlaid images, with increased contrast facilitating boundary identification. Rectangular grids were applied, tissue depth was measured at four regular intervals perpendicular to the epithelium, and average values were recorded (Figure 1). The corneal epithelium and CPD defects were measured similarly to each other, with a defect depth of 0 µm noted if no pink cells intersected the vertical lines. Depths were recorded in pixels and converted to µm using Image J scaling. Outcome measures included UVC penetration depth at the limbus based on CPD formation and counts of epithelial cells with and without CPDs.

### 2.6. Statistical Analysis

Data were checked for normality using the Shapiro–Wilk Test in GraphPad Prism (GraphPad Software, Boston, MA, USA; version 9), and appropriate tests were selected based on data distribution. A *p*-value < 0.05 was considered statistically significant.

## 3. Results

### 3.1. Depth of CPD Formation in the Porcine Corneal Epithelium

**Figure 2:**
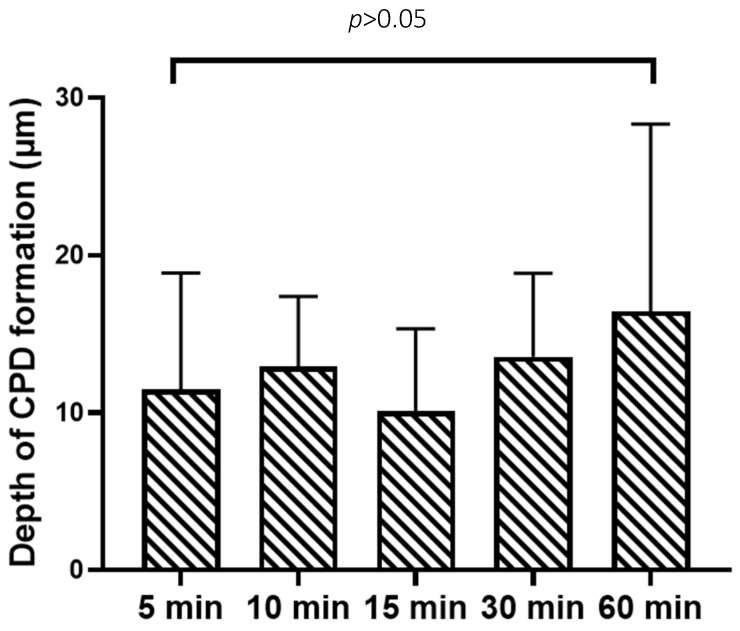
Estimation of the depth of CPD formation in porcine corneal epithelium (mean ± SD) after exposure to UVC (5 min to 60 min). Sample size was between 5 and 6 in each group.

**Figure 3:**
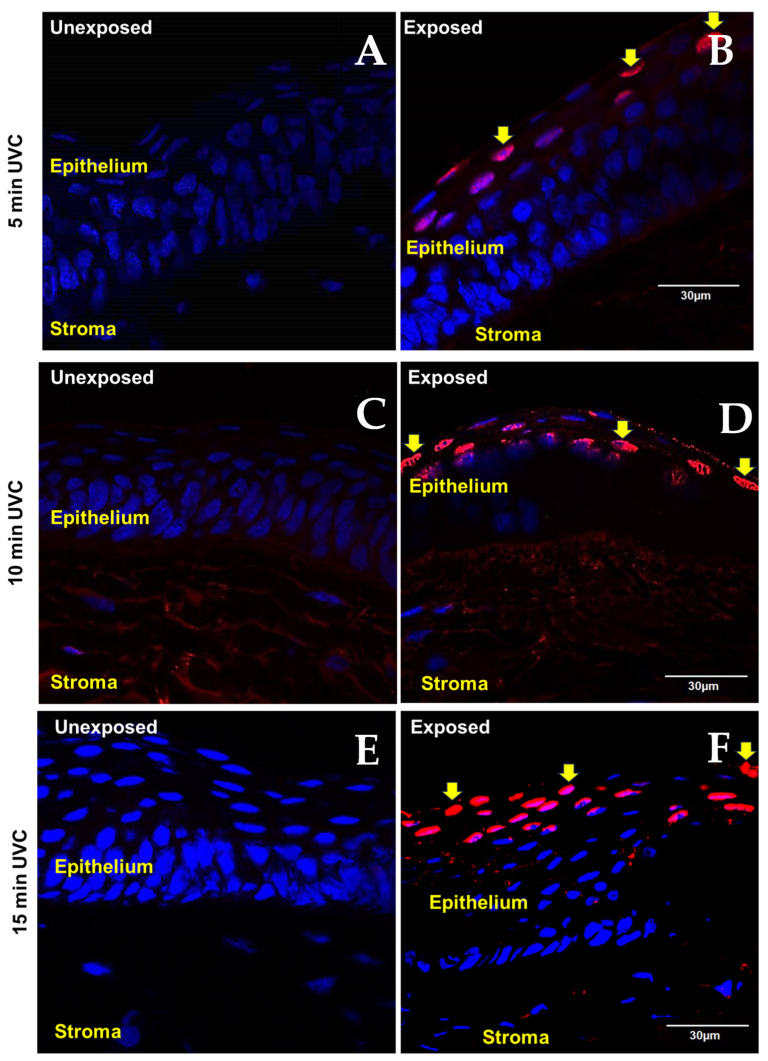

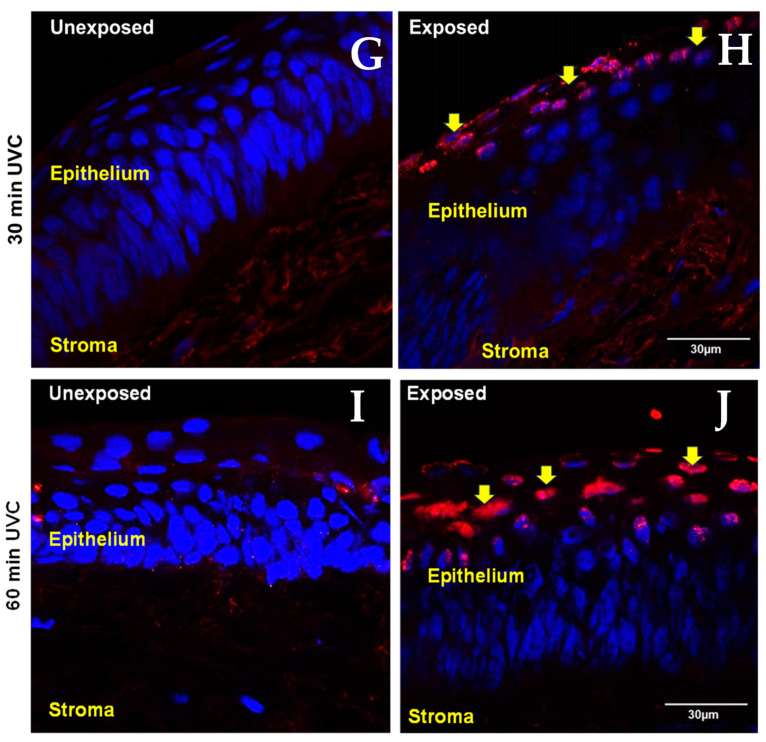
Representative images depict the status of the porcine cornea with respect to CPD formation following either no exposure (**A**,**C**,**E**,**G**,**I**) or varying durations of UVC exposure (**B**,**D**,**F**,**H**,**J**). CPD-positive cells, indicated by pink staining (yellow arrows), represent areas of DNA damage within a composite image of blue-stained nuclei and red-stained DNA lesions. CPDs were localised predominantly in the superficial layers of the corneal epithelium across all UVC exposure groups (5–60 min, sample size of between 5 and 6 per group). Comparable non-specific staining from the secondary antibody was observed in both exposed and non-exposed groups. Scale bar = 30 µm.

The total thickness of the epithelium in unexposed control groups and UVC-exposed groups is presented in Table 1. The epithelial thickness did not differ significantly between the control and UVC groups for any of the doses tested (*p* > 0.05). CPDs were detected only in the UVC-exposed corneas (Figure 2 and Figure 3).

The average depth of CPD formation (mean ± SD; % epithelial thickness) across the UVC exposure groups was 11.49 ± 7.38 µm (21.56 ± 12.63%; 95% CI: 7.82–15.16), 12.91 ± 4.47 µm (30.18 ± 29.70%; 95% CI: 8.22–17.60), 10.11 ± 5.21 µm (25.08 ± 15.04%; 95% CI: 7.10–13.12), 13.55 ± 5.29 µm (28.96 ± 15.12%; 95% CI: 9.12–17.98), and 16.45 ± 11.87 µm (28.66 ± 13.83%; 95% CI: 11.04–21.85) for the 5, 10, 15, 30, and 60 min exposures, respectively, with no significant differences found between the groups (*p* = 0.23, One-Way ANOVA) (Figure 2). The overall mean ± SD depth of CPD formation in the UVC-exposed groups was 13.3 ± 8.43 µm.

### 3.2. CPD Distribution in the Human Cornea

The human corneas sourced for immunohistochemistry analysis after exposure to UVC for either 1 min or 5 min or left unexposed for the same durations (sample size of 6 corneal sections per group, from three donors) did not support full epithelial cell analysis. Because the epithelial cell coverage in the corneal buttons was sparse and relatively thin, it was not possible to measure the depth of CPD formation in a comparable manner to that undertaken for the porcine eyes. The human tissue samples had been stored for an extended period prior to the study, as they were corneas preserved for transplantation, where the corneal rim becomes available for approved research use only after the central cornea is required clinically, for transplantation. As a result, the number of healthy cells and those with CPD-positive lesions were counted as a measure of the relative effects of UVC exposure (Table 2).

CPD-positive cells were detected only in the UVC-exposed groups and were confined to the superficial epithelium. The proportions of CPDs detected across the two groups are presented in Table 2. There was no difference between the proportions of CPDs between the 1 min and 5 min UVC exposures (*p* = 0.70).

## 4. Discussion

This study demonstrated that, for porcine corneas exposed to UVC for 5 to 60 min, CPD formation was confined to the superficial epithelial layers, to a depth of 13.3 ± 8.43 µm on average. Longer exposure times did not significantly affect CPD formation depth or density. Similarly, in human donor corneas exposed to UVC for 1 and 5 min, CPDs were restricted to the superficial epithelium. Previous studies have explored low-intensity UVC as a therapeutic option for superficial corneal infections [1,2]. Limbal stem cells differentiate into mature epithelial cells and migrate centripetally to renew corneal epithelium during a circadian turnover or corneal wound healing [14]. Therefore, the safety of limbal stem cells is important for corneal wound healing [15]. Significant insults to the cornea such as alkali burn can result in limbal stem cell failure and corneal opacity [6]. This study focused on whether UVC exposure could directly damage limbal stem cells [16,17] and also assessed the risk of limbal stem cell DNA damage given the potential carcinogenic risks of high or cumulative UV exposure [18]. The results indicate that narrowband UVC, when applied to a small, confined area in the cornea, does not reach the deeper layers where limbal stem cells reside [16,19], supporting the safety of these stem cells during UVC exposure.

This study utilised UVC-induced CPD formation as a measure of DNA damage [4,5,20,21,22,23]. Previous studies on UVC transmission through the cornea provided a physical measure of light penetration [2]. Combining both approaches, the safety of UVC was evaluated for potential clinical application. CPD formation and UVC depth penetration appeared to plateau by 5 min of exposure, with no significant increase in DNA damage or penetration depth beyond this time. These results align with those of Mallet and Rochette (2013), who observed similar findings in ex vivo human corneas, showing CPDs located primarily in the superficial epithelial layers, with no detectable damage beyond the first 10% of the stromal depth [24]. Our study confirms that UVC-induced host cell DNA changes occur within the most anterior one-third of the corneal epithelium.

The donor corneas used in this study were thinner than normal, with only one to two epithelial cell layers instead of the usual three to five, which is believed to be due to the storage process that involves one to two months of delay before use. Despite this, CPD formation remained confined to superficial layers. Cells with significant CPD formation typically undergo apoptosis, which, along with DNA repair, helps prevent mutagenicity [25]. In healthy individuals, the life cycle of epithelial cells is such that they are naturally shed from the superficial layers around every three days [26]. Given that UVC penetration is demonstrated to be limited to these layers during the short exposure to UVC (usually under 5 min), the risk of retaining DNA defects is unlikely, reducing the potential for cumulative DNA damage. The UVC doses tested—579 mJ/cm^2^ (5 min) and 6948 mJ/cm^2^ (60 min)—are within the range known to induce photokeratitis, which clinically presents with acute pain, tearing, and diffuse corneal staining, and the threshold dose for photokeratitis has been estimated to be approximately 5–10 mJ/cm^2^ [27]. Therefore, the doses used in this study were sufficient to cause epithelial damage.

A limitation of this study is the use of porcine corneas for investigating CPD formation following UVC exposure. While porcine and human corneas share important anatomical similarities, porcine corneas are generally thicker overall, with total corneal thickness reported to be approximately 1131 ± 87.5 µm in pigs [28] compared to around 550–565 µm in humans [29]. The difference in UVC exposure times was driven primarily by practical considerations and clinical relevance. Porcine eyes were readily available from a local slaughterhouse, allowing investigation of a broad range of exposure durations (5–60 min) to characterise the dose-dependency of the effects. In contrast, access to human donor corneas was limited. Therefore, in light of the porcine tissue outcomes, shorter, suprathreshold exposure durations (1–5 min) were selected, which still exceed but are closer in time frame to anticipated clinical doses, confirming the presence of UVC-induced effects while remaining clinically relevant. This approach allowed for a comprehensive exploration of exposure parameters in the porcine model and subsequent validation of key findings in human tissue.

Immunohistochemistry analysis revealed extensive non-specific secondary antibody binding to the corneal stroma arising from cross-reactivity relating to a porcine model and primary and secondary antibodies raised in mice and goats, respectively, as reported previously [30]. However, this cross-reactivity did not affect marker visualisation. The thicker porcine corneal epithelium may also introduce interspecies differences that could affect translation to the clinical setting [12]. Moreover, attempts to double label porcine stem cells, which would have allowed for the visualisation of intact cells, were unsuccessful. Consequently, although direct labelling of the stem cells was not achieved, it was possible to demonstrate that the deeper tissues within the limbal region, where stem cells reside, remained unaffected by CPD formation. This provides reassurance that stem cells within the deeper epithelial layers would be spared from damage during UVC exposure to the ocular surface. Another potential limitation is the lack of masking of images during analysis. However, the binary nature (Yes/No) of the assessment used for counting cells and the validated depth measurement protocol minimised subjectivity. The likelihood of bias is also minimal due to the mononuclear, readily identifiable and well-separated structural anatomy of the corneal epithelial cells.

While the current findings support the safety of UVC in terms of genotoxicity, the existing literature highlights other potential radiation-induced effects in the cornea. UVC in exceptionally high-dose exposure, such as that used in welding industries, has been linked to an increased risk of focal corneal opacities [31], possibly due to changes in corneal crystalline structure [32,33]. The present study evaluated the safety in terms of specific DNA damage, but there is a potential for other forms of cellular and functional damage, including cell apoptosis, sublethal cellular/tissue injury, or effects on protein structure and repair mechanisms. Therefore, further studies would be required to confirm UVC safety for keratitis management.

Additionally, preclinical studies report a slight reduction in keratocyte density after UVC exposure, similar to the effects seen after corneal collagen cross-linking, where keratocyte density decreases but returns to baseline within 6–12 months [34,35]. Given these considerations, further research on the impact of UVC on corneal proteins may be warranted to confirm its long-term safety in therapeutic application, balancing its benefits in treating corneal infections with any transient or non-transient effects on corneal health.

## 5. Conclusions

The study findings confirm that UVC-induced DNA damage in the cornea is limited to the superficial layers, with no detectable damage observed in deeper tissues where limbal stem cells reside, even after up to one hour of UVC exposure, far exceeding an anticipated therapeutic dose [1,2]. Combined with existing research, these findings provide strong evidence that corneal limbal stem cells may be spared from detectable CPD formation, supporting the potential safety of therapeutic low-intensity UVC application to the cornea.

## Figures and Tables

**Table 1:** Epithelial thickness (µm) across the UVC groups is expressed as mean ± standard deviation (95% confidence interval) for both control and exposure groups (sample size between 15 and 18). *p* < 0.05 is considered significant.

UVC Exposure Durations	Epithelial Thickness (µm)		
	Control Group	Exposed Group	*p*-Values (*t*-Tests)
5 min	50.07 ± 19.13 (35.37–64.77)	45.90 ± 16.03 (37.92–53.87)	0.58
10 min	38.28 ± 11.02 (30.40–46.16)	28.75 ± 9.95 (22.06–35.43)	0.05
15 min	42.09 ± 10.37 (36.10–48.07)	34.71 ± 11.35 (28.16–41.26)	0.08
30 min	45.33 ± 12.58 (39.26–51.39)	38.23 ± 10.87 (29.14–47.31)	0.15
60 min	50.61 ± 21.96 (39.70–61.53)	44.94 ± 22.52 (35.42–54.45)	0.41

**Table 2:** The proportions of CPDs within the epithelium are presented as mean ± standard deviation (95% confidence intervals) for both the control and exposure groups across the experimental conditions.

UVC Exposure Durations	Proportions of CPD (%)		
	**Control Group** **(sample size: 9–12,** **mean ± SD)**	**Exposed Group** **(sample size: 10–12,** **mean ± SD)**	**Exposure Comparison** ***p*** **-value** **(*t*-test)**
1 min	0.0 ± 0.0	37.2 ± 13.4 (28.75–45. 78)	0.0001
5 min	0.0 ± 0.0	39.05 ± 8.6 (32.90–45.21)	0.0001
Time comparison *p*-value (*t*-test)	-	0.70	-

CPD, cyclobutane pyrimidine dimers; SD, standard deviation; *p* < 0.05 is considered significant.

## Data Availability

Data are contained within the article.
